# The effect of climate change on the distribution of *Amblyomma hebraeum* and heartwater in South Africa

**DOI:** 10.1007/s11250-026-05197-1

**Published:** 2026-07-10

**Authors:** Magdaleen P. Wepener, Michiel M. Scholtz, Harold L. Weepener, Luis C. B. G. D. Neves

**Affiliations:** 1 Agricultural Research Council – Animal Production, Irene, South Africa; 2 Department of Veterinary Tropical Diseases, University of Pretoria, Pretoria, South Africa; 3 Department of Animal Science, University of the Free State, Bloemfontein, South Africa; 4 Agricultural Research Council - Natural Resources and Engineering, Pretoria, South Africa; 5 Centro de Biotecnologia, Universidade Eduardo Mondlane, Maputo, Mozambique

**Keywords:** Climate change, Habitat suitability, Heartwater, Modelling, Tick distribution

## Abstract

Historically *Amblyomma hebraeum* has been found in the north-eastern part of South Africa and along the coastal belt stretching to the Eastern Cape area. A change already noted in the past 20 years has been the increase in distribution area in the Eastern Cape, which now includes a larger inland area. It has been postulated that climate change has brought about a change in the distribution of *A. hebraeum* and subsequently, its potential tick-borne pathogens (TBP), *Ehrlichia ruminantium* and *Rickettsia africae*, in South Africa and that the effects of climate change may lead to further distribution changes in future. Habitat suitability modelling using Maxent software demonstrated that the distribution of *A. hebraeum* has altered compared to previous predictions done 15 years ago and that by the year 2065, most of the central and eastern parts of South Africa are predicted to have a high habitat suitability index for its presence. This indicates that the potential presence of diseases caused by *E. ruminantium* and *R. africae* would have to be considered in these previously unaffected areas when animals or humans show signs of illness. This expanded distribution of *A. hebraeum* and its TBP could have a substantial health and economic impact in South Africa.

## Introduction

Ticks of the genus *Amblyomma* transmit rickettsial diseases such as *Ehrlichia ruminantium*, the causative organism of heartwater in animals (Allsopp 2010), and *Rickettsia africae*, the causative organism of African tick bite fever in humans (Bitam 2012). The primary vector of these disease organisms in South Africa is *A. hebraeum*, the South African bont tick (Ledger et al. 2022; Walker and Olwage 1987). In the southern African region, 65% of all livestock are farmed within *Amblyomma* infested areas, with losses due to tick-transmitted diseases such as heartwater. In South Africa, the total estimated annual loss due to heartwater, including both direct and indirect losses (taken as cost associated with preventing and treating the disease), is approximately R1,266 million, with the direct losses amounting to 66.4% and indirect costs to 33.6% (van den Heever et al. 2022). Little is known about the effect of climate change on the distribution of *A. hebraeum*, and as a result that of *E. ruminantium* (causative organism of heartwater) and *R. africae* (causative organism of African tick bite fever in humans) in South Africa (Leask and Bath 2020b). Currently, there is an under representation of studies modeling the distribution of *Amblyomma* ticks and the rickettsial diseases that they carry compared to other tick-borne diseases (TBD) in the literature (Lippi et al. 2020). Most of the tick’s life cycle is in a non-parasitic condition (Norval 1973), and therefore the impact of climate change is an essential variable in tick control.

### Other studies on the effects of climate change on the distribution of *Amblyomma hebraeum*

Previous studies on climate change and the environmental suitability for *A. hebraeum* and *A. variegatum* that were conducted in Zimbabwe, found a good agreement between field records and modeled spatial range (Estrada-Peña et al. 2008). These studies also concluded that climate change could turn an unfavourable habitat into a favourable habitat niche for *A. hebraeum* within a few years (Estrada-Peña et al. 2008). It has also been shown that among the variables that affect the spread of African ticks, those affected by climate plays the most important role (Cumming 2002). The variables considered in making these observations were, a 6 × 6 degree grid, normalized difference vegetation index, vegetation type, elevation, rainfall, minimum and maximum temperature, and political regions. Factors affecting the distribution of *A. hebraeum* have been previously reported (Norval et al. 1994; Petney et al. 1987) and included soil moisture, air temperature, rainfall, altitude and vegetation types. Another potential factor is inter-species competition between *A. variegatum* and *A. hebraeum* (Bournez 2015). These parameters are discussed further under the heading of climatic conditions needed for the survival of *A. hebraeum.* A survey conducted amongst veterinarians and farmers found that there was general agreement that the area in which *E. ruminantium* occurred had changed compared to historical records (Leask and Bath 2020 a).

### Climate change in South Africa

Over the period of 1960 to 2012 the mean average temperature in South Africa has increased by 1.4 °C, of which the most marked increase occurred in the Western and Northern parts of the country (DEA: Full technical report on climate trends and scenarios of South Africa). Annual rainfall has stayed relatively consistent, but more dramatic rainfall events and increases in dry or wet periods have been reported in some areas (Kruger 2006). One notable change in the timeframe of 1960–2010 was a decrease in rainfall in the central interior of the country (MackKellar et al., 2014). A historic trend in rainfall measured during the period of 1910–2004 showed a decreased rainfall in northern Limpopo, an area of northeastern Free State, western Kwazulu-Natal, southern Mpumalanga, two areas of southeastern Eastern Cape and the South Coast of South Africa (Kruger 2006).

It is estimated that in the next three decades the temperature will increase by an average of 1.5–2.0 °C, with most of the southern Africa region showing a reduced rainfall, except for the central interior and Eastern Cape regions in South Africa that will have a wetter rainfall season. The areas predicted to have the greatest decrease in rainfall are the eastern part of Limpopo, Mpumalanga, south west- and southern Cape regions (Meissner et al. 2014).

### Description *A. hebraeum*

Ticks belonging to the genus *Amblyomma* are typically large Ixodid ticks with long anterior mouthparts, eyes, rings on the legs and enamelled ornamentation on the scutum (females) or conscutum (males). The males and females have festoons, but the festoons are not very visible in fully fed females. *A. hebraeum* is identified to species level based on its flat, very marginal eyes, the presence of small to medium punctations evenly spaced on the scutum/conscutum and a unique complex enamelled ornamentation. The distinctive elaborate enamel on the middle line of the male festoons range from pink to orange, while the marginal festoons have no elaborate enamel and are plain reddish-brown (Walker et al. 2014).

*A. hebraeum* belongs to the family *Ixodidae* and this is one of the families that has four stages in their life cycle, namely, egg, larva, nymph and adult (Horak et al. 2002 b). *A. hebraeum* is a three-host tick meaning that at each stage of the lifecycle the tick drops off the host to moult and develop further in the environment before questing to find a blood meal from a new host (Leal et al. 2020). Eggs hatch on the ground and larvae wait on vegetation for a host to pass by. The six-legged larvae attach and feed for 7–14 days before detaching to moult in the environment. Nymphs and adults are active hunters, activated by CO_2_ exhaled by the host animal (Norval et al. 1989). Nymphs will again attach, feed for 7–14 days and detach to moult (Walker et al. 2014). Adult males secrete a pheromone which attracts adult females and nymphs to attach in their close proximity (Norval et al. 1989). Males of *A. hebraeum* do not complete spermatogenesis until they have had a blood meal, and females have been found not to attach without the presence of males that have been attached for close to a week (Norval 1973). Females will feed for 7–9 days after mating with the male before dropping to the ground where she can lay up to 20,000 eggs (Walker et al. 2014).

Hosts include domestic ungulates (domestic bovine, porcine, ovine and caprine animals), wild animals and birds, with smaller hosts tending to have more immature ticks on them (e.g. guinea fowl, hares, duiker) and the larger hosts (e.g. cattle, eland, buffalo) having more adult stages (Horak et al. 1987). In areas where domestic ungulates are absent or frequently dipped, the role of wild animals as hosts becomes more important (Jensenius et al. 2003 b).

### Climatic conditions for the survival of *A. hebraeum* in South Africa

As *A. hebraeum* is a three-host tick, it spends 90% of its life cycle in the environment, not attached to a host (Norval 1973). This makes environmental factors critical for the survival of the species. Environmental factors that affect survival of the tick, especially the desiccation of the larval stages, include relative humidity, temperature, rainfall, seasonality, natural predators, photoperiod and habitat (Leal et al. 2020).

*A. hebraeum* is found in wooded areas and does not occur in treeless grassland. It is predominant in coastal bush, riparian woodland, thornveld and Mopani woodland (Jongejan et al. 2020). The reason for this is most likely the sensitivity of the eggs to desiccation. The temperature and humidity required for survival of the different stages of *A. hebraeum* vary, with later stages being more resistant to temperature and humidity fluctuations. Pre-oviposition females will die at 15 °C, with a humidity of 40% after two months. The optimum temperature for oviposition and egg development is 20–30 °C, 26–35 °C for nymphs, and 20–35 °C for the pre-moult adult stages. In general eggs are not oviposited in hot open grassland or waterlogged areas, whereas the few eggs that are laid, do not hatch (Norval 1977). *A. hebraeum* also favours areas with a dry period between April and August (Estrada-Peña et al. 2008). An optimal rainfall range of 300–800 mm and an altitude of 0–1,525 m above sea level (Petney et al. 1987) has been suggested for this species.

### Historical and current geographical distribution of *A. hebraeum* in South Africa

Historically *A. hebraeum* occurred in the north-eastern part of South Africa and in the coastal belt area up to the Eastern Cape area (Walker et al. 2014). In the last twenty years, the distribution in the Eastern Cape has been noted to be more inland than previously recorded (Horak et al. 2009). There have also been reports of *A. hebraeum* being established in the Free State area of Boshoff (Horak et al. 2015). Goats tested positive for *E. ruminantium* antibodies in the Northern Cape, Northwest, Limpopo, KwaZulu-Natal and the Eastern Cape provinces of South Africa. However, some of these animals may have been imported from other provinces or infected with non-pathogenic strains of *E. ruminantium*, (Mdladla et al. 2016).

**Fig. 1:**
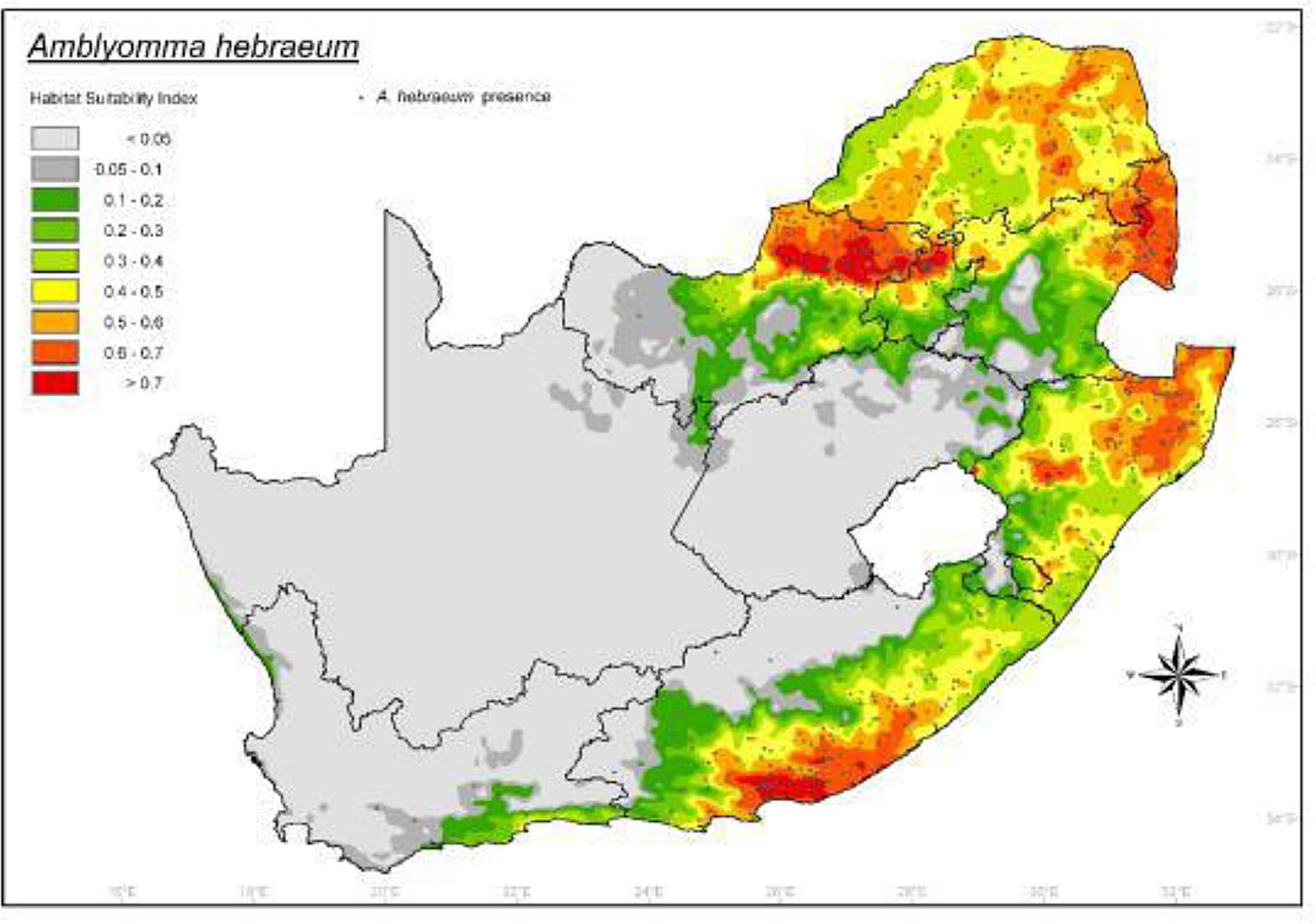
Habitat suitability for *A. hebraeum* as estimated in 2008 (Spickett, personal communication)

In addition to the literature review distribution points, coordinate data from a 2008 study by Spickett and colleagues was made available to use through personal communication as a reference for this study. Their habitat suitability model for *A. hebraeum* is shown in Fig. 1.

### Changes in land use and its impact on the distribution of *A. hebraeum*

Land use affects the prevalence of ticks as the interaction between the tick and its environment has two requirements, vertebrate hosts for blood meals and a suitable micro environment when they are not on the hosts (Ledger et al. 2019). In some areas such as the Eastern Cape, a change from livestock to game farming has led to an increase in the livestock wildlife interface. This interface has led to an increase in tick prevalence and diversity, with more ticks and tick species found in grazing areas where wildlife and livestock are kept in close proximity to each other, compared to predominant cattle areas (Smith and Parker 2010). Furthermore, vegetation on game farms is normally less controlled than on cattle farms. Grass is left to grow taller and bush encroachment is not as controlled. This might lead to a better-suited microclimate that protects ticks from desiccation (Smith and Parker 2010).

Due to the fact that climate change has been proposed as a reason for the wider spread of *A. hebraeum* in South Africa, this study investigated the possible effect of climate change on the distribution centred around the period of 2005 and projected the distribution in 30 year increments up to 2065 of *E. ruminantium* and *R. africae* due to the potential change in the distribution of the vector, *A. hebraeum* in South Africa.

## Materials and methods

Ethical clearance was obtained from the Research Ethics Committee and Animal Ethics Committee of the University of Pretoria, Onderstepoort campus (REC167-21).

A qualitative literature review was conducted to identify the climatic conditions that were most favourable for *A. hebraeum* survival and to determine the historical and present geographical distribution of *A. hebraeum*. Data was collated from the Agricultural Research Council meteorological data sites. Quantitative survey studies were used to assess the distribution of *A. hebraeum* to new areas. All of this data was then used to model the potential change in distribution of *A. hebraeum* due to climate change using Maxent software (Williams et al. 2016).

### Current data (1991 to 2020)

Information on current weather data was collated from the Agricultural Research Council (ARC) weather stations. The ARC Weather Station Network consists of some 520 active automatic weather stations (Agrometeorology Staff 2022). In addition, data records of about 1,400 historic stations are hosted in the information system. Hourly data measured at the automatic weather stations include air temperature, rainfall, relative humidity, wind direction, wind speed and solar radiation. From these recordings, daily values are calculated for maximum temperature (Tx), minimum temperature (Tn), daily rainfall, maximum relative humidity (Hx) and minimum relative humidity (Hn).

The weather station data was used to prepare monthly interpolated surfaces for the period of January 1991 to December 2020 for total monthly rainfall as well as average Hn, Hx, Tn and Tx. Long-term averages were calculated for each month resulting in 12 surfaces for each of the variables.

### Climate projections

Six different dynamically downscaled Coupled Global Climate Model (CGCM) projections of future climate change (Engelbrecht et al. 2011) were used. The regional model used is the Conformal-Cubic Atmospheric Model (CCAM), a variable resolution global atmospheric model of the Commonwealth Scientific and Industrial Research Organization (CSIRO) in Australia (McGregor 2005). The model was applied in stretched-grid mode over southern and tropical Africa, to obtain simulations at a resolution of approximately 0.5° in longitude and latitude. All the CGCM simulations are for the A2 Special Report on Emissions Scenarios (SRES) and were downscaled for the period 1961–2100.

The daily CGCM data was summarized into monthly rainfall totals, monthly average of maximum temperatures and monthly average of minimum temperatures. The monthly data was in turn averaged over 30-year periods, centred around the following years: 2005, 2035, and 2065. For example, the average rainfall for January centred around 2035 would be the average of rainfall in January from January 2021 to January 2050 for each of the six CGCMs. Median values of the six CGCMs were used to obtain one set of variables centred around the periods 2005, 2035 and 2065.

Finally, the median values of changes from 2005 to 2035 and from 2005 to 2065 of six different dynamically downscaled CGCM projections were applied to the long-term average surfaces that were derived from the ARC weather station network, resulting in the following datasets:

- **Dataset 1**: Monthly long-term average data (Rainfall, Hn, Hx, Tn and Tx) centred around 2005 interpolated from the weather station data (1991–2020).
- **Dataset 2**: Monthly long-term average data (Rainfall, Hn, Hx, Tn and Tx) centred around 2035 (2021–2050) by applying the median values of changes from 2005 to 2035 of the six CGCMs to dataset 1.
- **Dataset 3**: Monthly long-term average data (Rainfall, Hn, Hx, Tn and Tx) centred around 2065 (2051–2080) by applying the median values of changes from 2005 to 2065 of the six CGCMs to dataset 1.

Annual statistics that were derived from the monthly long-term average surface values (centred around 2005, 2035 and 2065) for usage in Maxent are presented in Table 1. The following variables were assumed to be static since no future data are available: elevation, nine vegetation biomes and 35 bioregions. Information on the variables - average annual maximum relative humidity and average annual minimum relative humidity were not available for the future climate models that were used. These five variables were therefore kept the same for future periods.

**Table 1:** Statistics derived from the monthly long-term average surface values that was used in Maxent

2005	2035	2065
Average annual rainfall Average maximum temperature of the warmest month Average minimum temperature of the warmest month Average maximum temperature of the coldest month Average minimum temperature of the coldest month Average annual maximum relative humidity Average annual minimum relative humidity Elevation Nine vegetation biomes 35 Bioregions	Average annual rainfall Average maximum temperature of the warmest month Average minimum temperature of the warmest month Average maximum temperature of the coldest month Average minimum temperature of the coldest month	Average annual rainfall Average maximum temperature of the warmest month Average minimum temperature of the warmest month Average maximum temperature of the coldest month Average minimum temperature of the coldest month

The elevation was derived from the digital elevation model, as prepared by Weepener et al. (2012). Mucina and Rutherford (2006) described and mapped 440 zonal and azonal vegetation types, 35 bioregions and nine vegetation biomes for South Africa, Lesotho and Swaziland.

All data were projected to the Albers Equal-Area projection with Central meridian: 24 ºE, first standard parallel at 24 ºS, and second standard parallel at 33 ºS. The data was resampled to 1 km grid cells with 1,647 columns and 1,415 rows.

### Maxent analysis

Maximum entropy (Maxent) is a machine learning modelling technique used to make predictions or inferences. It is used in diverse areas such as astronomy, statistical physics, and signal processing (Phillips et al. 2006). Recently it has been widely used as a general approach to modelling species distribution with presence-only (PO) data points. It estimates the less constrained distribution of training points compared with random background locations, with environmental data layers defining constraints (Baldwin 2009).The results illustrate how well the model fits the location data compared with random distribution (Phillips et al. 2006). This model has been used by (Williams et al. 2016) to identify suitable (optimal) geographical areas for milk production in Holstein herds on pasture with geographical locations of the farms used as PO data points. The same modelling technique may be used to predict the future distribution of ticks, TBD and other vectors and associated diseases, as was done in Zimbabwe for *A. hebraeum* and *A. variegatum* (Estrada-Peña et al. 2008).

In order to use Maxent for analysis in the current study, it was necessary to ensure that the grids were all identical as pertaining to the raw data that was used. To do this, the data were first plotted in ArcGIS. GPS coordinates of tick occurrence points as supplied by A.M. Spickett, and the new collection sites were used as the current occurrence points for *A. hebraeum.* Environmental layers were then added to include humidity, temperature, elevation, biomes, bioregions and rainfall. Maxent was set to use automatic features, to use 25% of the occurrence points for random testing and 75% for training. The maximum iterations used was set as 500, the default prevalence was set at 0.5 and the threshold rule used was minimum training presence. A replicate run was set for cross validation. Four separate runs were done. In scenario 1, all the environmental layers were added even though no references for future values of humidity, biomes, and bioregions were available. In scenario 2, only the layers for which future data was available were used, e.g. temperature and rainfall. In scenario 3, all the layers except for humidity were used as it was inferred that humidity depends on the rainfall and temperature variables and thus already accounted for. In scenario 4, all the layers for which future predictive data was available were used, as well as elevation as that would not change.

## Results

### Climatic data

**Fig. 2:**
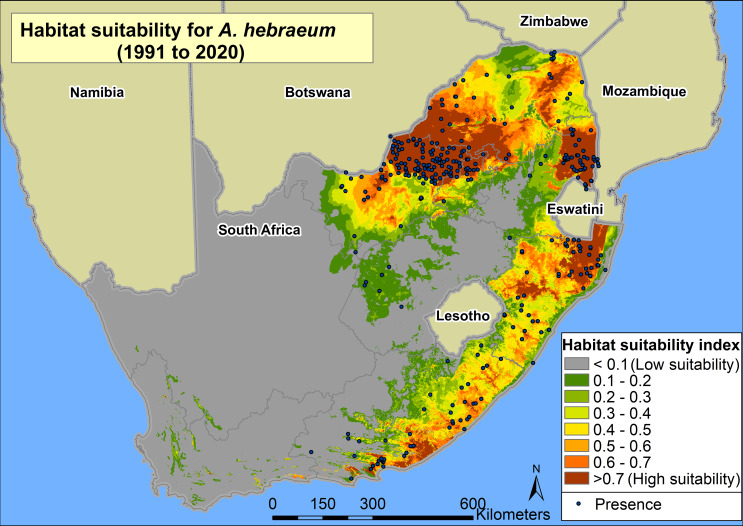
Habitat suitability index for around 2005 (1991–2020) with all the variables (as indicated in Table 1)

**Fig. 3:**
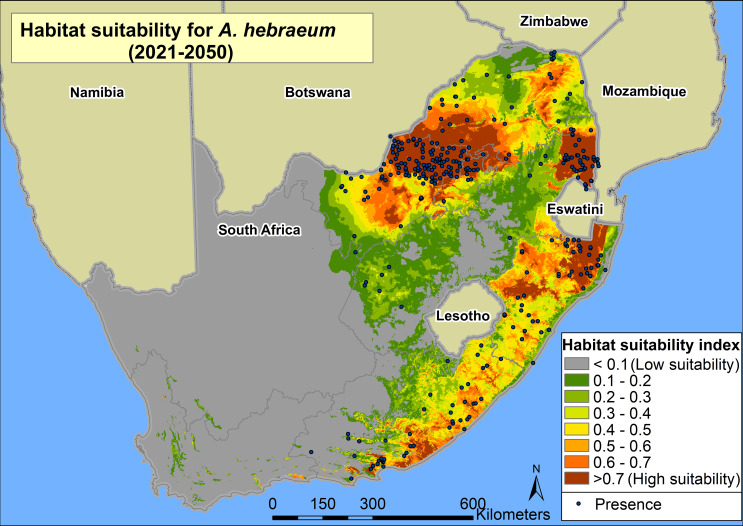
Habitat suitability index for around 2005 (2021–2050) with all the variables (as indicated in Table 1)

**Fig. 4:**
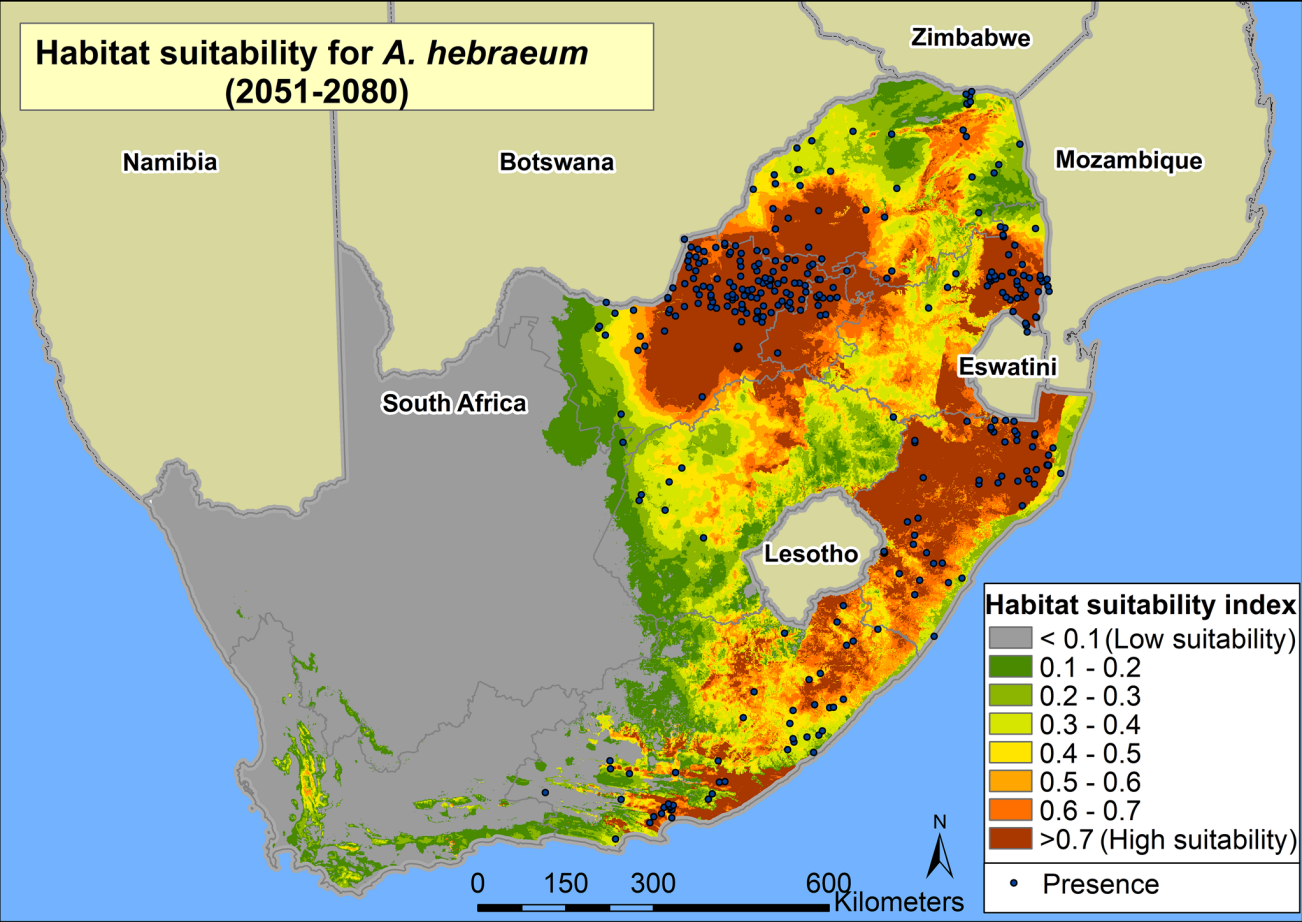
Habitat suitability index for around 2065 (2051–2080) with all the variables (as indicated in Table 1)

The data for current and future *A. hebraeum* distribution as predicted by Maxent is illustrated in map format. Occurrence points for *A. hebraeum* are shown on each map. The features chosen by the automation were linear and quadratic features and were based on the sample size (occurrence points). Scenario 1, which includes all the variable layers is depicted in Figs. 2, 3 and 4. Figure 2 shows the current habitat suitability for *A hebraeum* centered around 2005 (1991–2020), Fig. 3 shows the increased area of habitat suitability for *A. hebraeum* around 2035 (2021–2050) and Fig. 4 shows the largest area of habitat suitability for the tick around 2065 (2051–2080). The colours start as grey, that indicates they are not suitable and then it moves through the cooler (lighter) colours to the warm (darker) colours, which is red, that has the highest probability of suitability for the tick.

Across all the models, the variable (layer) that contributed the most to the distribution of *A. hebraeum* was rainfall, followed by biomes and bioregions when they were included. To prove the validity of the predictions, the statistical testing of omission rate versus the predicted omission can be used as a function of the cumulative threshold for the threshold-dependent binomial omission test and the Area under the curve (AUC) as the threshold-independent test.

The 25% of samples that were used for statistical testing show that the omission on the test and training samples closely follow the predicted omission line for all scenarios at both future prediction times. The Receiver operator characteristics curve showed an AUC for the test samples of between 0.849 and 0.822 for all samples.

## Discussion

When comparing the current habitat suitability map (Fig. 2) to the predicted maps (Figs. 3 and 4), it can be seen that the area of habitat suitability for *A. hebraeum* will definitely change and have already changed from what was previously predicted by other authors, as seen in Fig. 1.

Areas of increased habitat suitability can especially be seen in the central part of South Africa. Regardless of the environmental layers that were added or omitted, the prediction for 2035 and 2065 still showed this trend of habitat suitability, where the 24.5-degree meridian almost divides the country into an eastern area where *A. hebraeum* occurs and a western area where it is absent. Interestingly, the most northern part of South Africa as well as an area just northeast of this shows a decrease in habitat suitability for *A. hebraeum*. It would be interesting to validate this trend through future tick collection. The present results indicate that Lesotho would likely remain mostly free of the tick and this might be due to the high elevation of this mountainous country.

According to the models of this study, the variable that will have the most effect on the distribution of *A. hebraeum* is rainfall or the absence thereof, which will especially affect the desiccation of the egg and larval stages of the tick. This can be expected as desiccation of the eggs especially has always been noted as a limiting factor for tick survival (Norval 1977). As previously discussed, the central interior of South Africa is expected to show an increase in the amount of rainfall in the next 30 years (Meissner et al. 2014), and this relates to the eastern part of the country where the prediction models show an increase in habitat suitability. From meteorological data collected, it is known that there is a northern Limpopo area that has shown decreased rainfall for the period 1910–2004 (Kruger 2006), and this area also shows a decreased habitat suitability in the current predictions.

The second most important variable was biome. The vegetation and climate in a large area would be described as part of the specific biome. A suitable biome would provide the female ticks with an area to lay the eggs that are not too waterlogged or too dry leading to desiccation. These also provide areas for ticks that have dropped off from their hosts to survive adverse weather conditions such as cold and drought spells and provide vegetation for the larval and nymph stages to quest on for passing hosts.

The current model was proven to be statistically valid based on testing using the threshold dependent as well as the threshold independent test. This study’s threshold dependent evaluation is an extrinsic omission rate, indicating how many of current test points fall outside the predicted habitat suitability (Phillips et al. 2006). A low omission rate is necessary for a good model (Anderson et al. 2003). This study’s omission line closely followed the predicted omission line and the test omission rate stayed below 2.5%, so it can be concluded that the data is valid.

As a threshold independent evaluation, the Receiver Operator Characteristics (ROC) was used. The ROC curve showed an area under the curve for the test samples all above 0.8, meaning that this study’s test results were between 0.8 and 0.9 and therefore provide an excellent representation of what is really happening (Mandrekar 2010).

The prediction of habitat change by various models has been developing steadily through the last couple of decades. The best form of evidence for climate change is showing that climate change has occurred in conjunction with the change in habitat for the tick (Ogden et al. 2021). As previously stated, the fact that Ixodid ticks spend most of their lives in the environment rather than on the host, makes them extremely sensitive to changes in the climate as well as changes to their micro-environment, that will be altered through external factors such as the change in land usage from cattle farming to wildlife farming.

## Conclusions and recommendations

Overall, the findings of the present study indicate that climate change has a notable effect on the distribution of *A. hebraeum.* The value of the use of Maxent as a model for the prediction of future distribution of *A. hebraeum* seems to be very high. The variable that was found to contribute the most to the predicted change in *A. hebraeum* distribution pattern was rainfall.

This study shows that habitat suitability has definitely changed for *A. hebraeum*, making it possible for the tick to survive in areas where it had not previously been found. Additionally, the area of habitat suitability is predicted to increase in the future, especially in the eastern part of the country, spreading westwards. The spread of pathogens carried by a tick vector can always be related to the spread of that tick, especially with uncontrolled movement of animals from areas where these diseases are present. It can thus be concluded that the spread of *E. ruminantium* and *R. africae* will likely move across the whole eastern part of the country towards the west, following the distribution of *A. hebraeum.*

These results are valuable to farmers, government departments and veterinarians in planning timely prophylactic measures and control strategies in order to decrease the debilitating impact of heartwater in their communities and on their farms. In addition, the well documented direct traumatic effect due to tick bites may be minimized.

It is recommended that farmers should be educated to look out for the ticks as well as to look for symptoms of the diseases caused by these ticks to prevent livestock losses. Good biosecurity and attention to tick control on any animals bought from especially endemic areas need to be rigorously applied. More research into the potential adaptation of the tick to survive in different climatic conditions will help to refine the future predictive value of niche habitat suitability modelling.

## Data Availability

The data that support the findings of this study is available from the first author (M.P. Wepener), upon reasonable request.
